# A linear/quadratic order parameter coupling description of the Verwey transition in magnetite, Fe_3_O_4_

**DOI:** 10.1107/S2052520625004779

**Published:** 2025-07-08

**Authors:** Michael A. Carpenter, Richard J. Harrison, James Shaw-Stewart, Kanta Adachi, Mark S. Senn, Christopher J. Howard

**Affiliations:** a Department of Earth Sciences University of Cambridge Downing Street CambridgeCB2 3EQ United Kingdom; b Everoze Partners Ltd, Lancaster Environment Centre Lancaster University LancasterLA1 4YQ United Kingdom; c Graduate School of Engineering Osaka University 2-1 Yamadaoka Suita Osaka Japan; d Department of Chemistry University of Warwick CoventryCV4 7AL United Kingdom; e School of Engineering, University of Newcastle, University Drive, Callaghan, New South Wales2308, Australia; Academy of Sciences of the Czech Republic, Czechia

**Keywords:** magnetite, Verwey transition, charge order, order parameter coupling, spontaneous strain

## Abstract

The change in symmetry *Fd*3*m* → *Cc* at the Verwey transition in magnetite puts it in a class of phase transitions with linear/quadratic coupling between two separate order parameters. Direct coupling between an order parameter *Q*_e_, to represent an electronic instability, and an order parameter *Q*_co_, to represent cation charge ordering, has the form λ*Q*_e_*Q*_co_^2^, with *T*_c_(*Q*_e_) < *T*_c_(*Q*_co_), but there must also be indirect coupling through the common strain, *e*_6_, due to strain coupling terms λ*e*_6_*Q*_e_ and λ*e*_6_*Q*_co_^2^. *Q*_e_ has the symmetry of irrep Γ_5_^+^ while the pattern of cation charge ordering of Fe^2+^ and Fe^3+^ on octahedral sites depends on some combination of irreps Δ_5_, X_1_, X_3_, W_1_ and W_2_. The software package *ISOVIZ* has been used to show how reported patterns of order for the simplified structure in space group *P*2/*c* can be understood in terms of a linearly dependent mix of patterns with symmetry Δ_5_ and X_1_, so that *Q*_co_ can be treated in the first instance as though it has the symmetry of Δ_5_. Spontaneous strains calculated from published lattice parameters and symmetry-adapted atomic displacements from previous structural refinements in space group *Cc* have been used to confirm that the two order parameters have different temperature dependences, consistent with this phenomenological treatment. The effect of chemical doping can be understood in terms of the development of local strain heterogeneity which acts to suppress the macroscopic strains and which appears to have a greater influence on charge ordering than on the electronic structure.

## Abstract 2

The change in symmetry at the Verwey transition in magnetite puts it in an important class of phase transitions with linear/quadratic coupling between two separate order parameters. We use group theory to characterize the form of the coupling between charge order and an electronic instability, and test the model with structural data.

## 1. Introduction

The Verwey transition in magnetite, Fe_3_O_4_, has attracted intense interest since the first report of a large, discontinuous change in resistivity at *T*_v_ ∼125 K (Verwey, 1939 ▸). Recent structural studies have resolved many of the issues relating to the driving mechanism for the transition by confirming that it involves a combination of electronic effects, including Jahn–Teller distortion of sites containing Fe^2+^, with charge ordering such that Fe atoms with different nominal charges adopt an ordered arrangement on the octahedral sites (Wright *et al.*, 2001 ▸, 2002 ▸; Goff *et al.*, 2005 ▸; Senn *et al.*, 2012*a* ▸, 2012*b* ▸, 2013 ▸, 2015 ▸; Perversi *et al.*, 2016 ▸; Attfield, 2022 ▸). Below *T*_v_ the ordered structure is in crystallographic space group *Cc* with lattice parameters 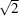
*a* × 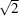
*a* × 2*a*, where *a* represents the cell dimension of the parent cubic structure (Iizumi & Shirane, 1975 ▸; Yoshida & Iida, 1979 ▸; Iizumi *et al.*, 1982 ▸; Senn *et al.*, 2012*a* ▸; Perversi *et al.*, 2016 ▸). The complex electronic structure has been found to involve highly structured three state polarons known as trimerons (Senn *et al.*, 2012*a* ▸, 2012*b* ▸, 2015 ▸; Perversi *et al.*, 2016 ▸; Attfield, 2022 ▸).

Rather than dwelling on these details, the purpose of the present study was to consider the Verwey transition from a different perspective, as an important case of linear/quadratic coupling between two order parameters with different symmetries. This form of coupling has specific consequences for the evolution of crystals with multiple instabilities (Cano *et al.*, 2010 ▸; Salje & Carpenter, 2011 ▸), as has been shown for electronic and magnetic order parameters in Ba(Fe_1–*x*_Co_*x*_)_2_As_2_ (Cano *et al.*, 2010 ▸; Böhmer & Meingast, 2016 ▸; Carpenter *et al.*, 2019 ▸), NdCo_2_ (Driver *et al.*, 2014 ▸), and Pr_0.48_Ca_0.52_MnO_3_ (Carpenter *et al.*, 2010*a* ▸, Carpenter *et al.*, 2010*b* ▸), for structural and magnetic order parameters in Fe_1–*x*_O (Carpenter *et al.*, 2012 ▸) and for electronically driven transitions with multiple order parameters in alloys with martensitic transitions such as Ti_50_Pd_50–*x*_Cr*_x_* (Driver *et al.*, 2020 ▸) and the Heusler compound Ni_50+*x*_Mn_25–*x*_Ga_25_ (Salazar Mejía *et al.*, 2018 ▸).

We make use of the software suite *ISOTROPY* from Brigham Young University (Stokes *et al.ISOTROPY* Software Suite, https://iso.byu.edu) to identify the symmetry properties of two order parameters. One of these, *Q*_e_, is used to represent purely electronic aspects of the transition and a second, *Q*_co_, to represent cation charge ordering. Following other group theoretical treatments (Piekarz *et al.*, 2007 ▸), we focus on the role of the linear/quadratic coupling term 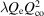 in a Landau expansion, where λ is a coupling coefficient. *ISOVIZ* from the *ISOTROPY* package is used to illustrate the possible patterns of cation charge order that might be represented by *Q*_co_.

As a test of this description of the transition, lattice parameter data from the literature (Wright *et al.*, 2000 ▸; Senn *et al.*, 2015 ▸; Pachoud *et al.*, 2020 ▸) are used to follow the temperature dependence of spontaneous strains which couple with each of *Q*_e_ and *Q*_co_. Symmetry-adapted atomic displacement data obtained using *ISODISTORT* and reported by Senn *et al.* (2015 ▸), are used to explore relationships between order parameters with different symmetries that relate to the cation charge ordering.

## 2. Symmetry analysis

Magnetite, Fe_3_O_4_, at room temperature is an inverse spinel, that is the structure is cubic in space group *Fd*3*m*. Tetrahedral sites, Wyckoff 8*a*, nominally contain eight Fe^3+^ while octahedral sites, 16*d*, nominally contain eight Fe^2+^ + eight Fe^3+^. *Fd*3*m* symmetry evidently requires that Fe^2+^ and Fe^3+^ have a disordered arrangement on the octahedral sites. In order to respect developments in the literature and to illustrate a simpler case first, we follow Yamauchi *et al.* (2009 ▸) in initially treating the transition as being from *Fd*3*m* to *P*2/*c* and then considering the refined structure in space group *Cc*.

### *P*2/*c* (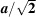 × 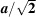 × 2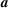)

2.1.

Piekarz *et al.* (2007 ▸) showed that no single order parameter can yield a structure in space group *P*2/*c* with unit cell 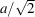 × 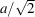 × 2*a*, where *a* is the lattice parameter of the parent cubic structure. A combination of order parameters with the symmetries of at least two irreducible representations (irreps) of space group *Fd*3*m* is required to achieve this reduction in symmetry. The full list of irreps is given in Table 1 of Piekarz *et al.* (2007 ▸) and in Table S1 of the supporting information that goes with the present paper. Pairwise combinations of irreps that can give the required symmetry change are listed in Table S1. As a requirement of symmetry, the transition must be first order in character.

Evidence of which two irreps to choose is provided initially by softening of the single crystal elastic modulus *C*_44_ as *T*_v_ is approached from above. This is based on softening reported for magnetite by Schwenk *et al.* (2000 ▸) and Kozłowski *et al.* (2000 ▸) which is characteristic of bilinear coupling between the strain component *e*_4_ and an order parameter with symmetry 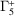. The atomic scale mechanism could, in principle, involve a classical soft acoustic mode, an electronic instability or some combination of the two. For present purposes the essential point is that it has the symmetry of 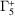. (Note that, for the benefit of readers who may not be so familiar with group theory, we use the informal description ‘has the symmetry of’ in place of the more formal expression ‘transforms as’.)

The symmetry of the second order parameter must account for the pattern of cation charge order. Out of the full list of irreps given in Table S1, only 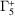, Δ_5_, X_1_, and X_3_ lead to ordering in the octahedral sites of the *P*2/*c* structure. The patterns of ordering for each of these have been generated using *ISOVIZ* and are illustrated using different sized circles in Figs. S1–S4 in the supporting information for the succession of eight layers within the unit cell (*z* = 0, 1/8*c*, 2/8*c*, 3/8*c*, …). Each illustration shows three different sized circles, some increased in size, some reduced in size and some unchanged by the symmetry operator.

**Figure 1:**
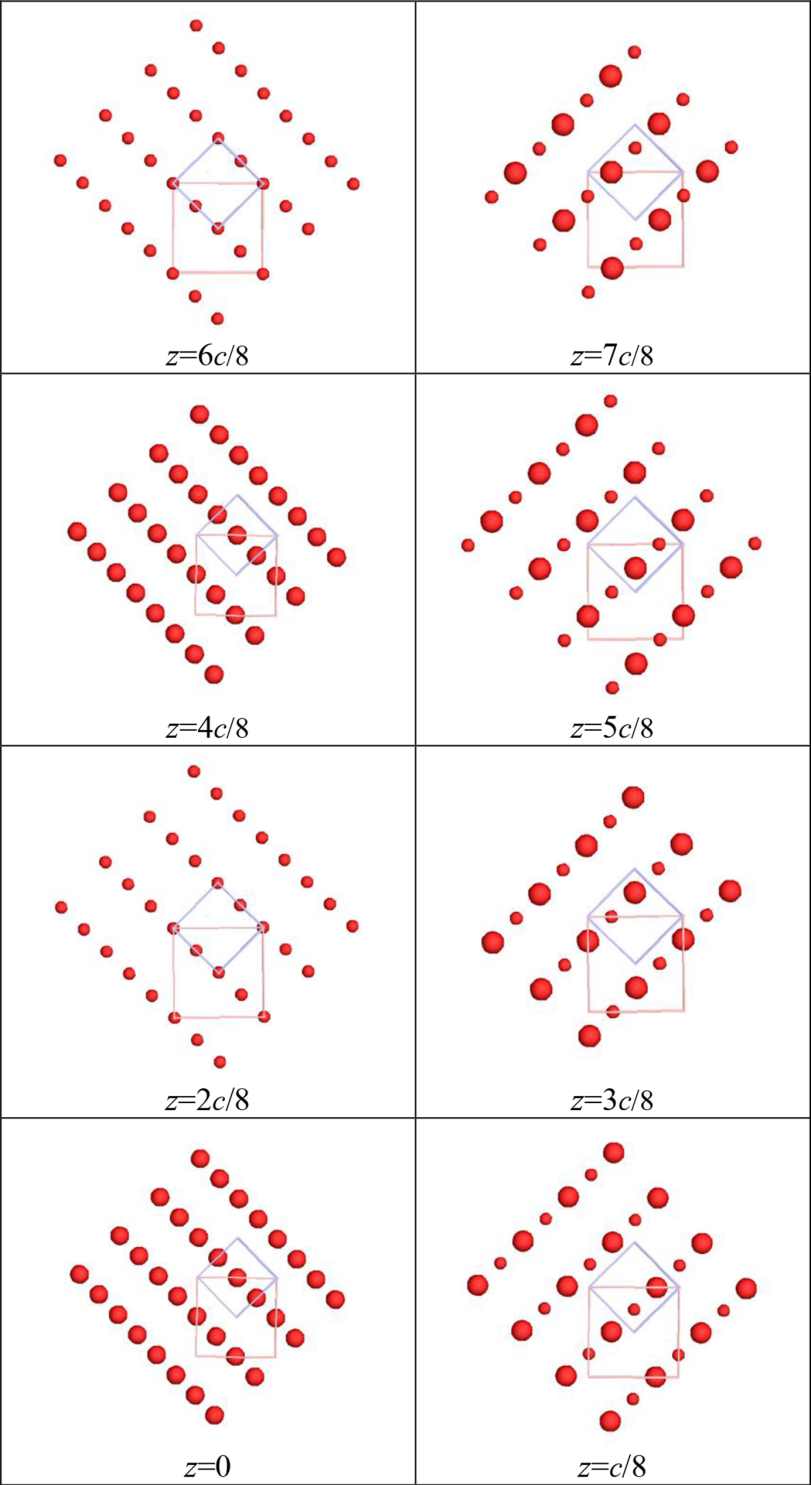
Schematic representation of cation charge ordering in octahedral sites for ordered arrangements arising from equal proportions of Δ_5_ and X_1_. Small and large circles represent two different formal charges. Δ_5_ gives alternation in rows within the layers at *z* = *c*/8, 3*c*/8, 5*c*/8, 7*c*/8, but has no effect on atoms in the layers at *z* = 0, 2*c*/8, 4*c*/8, 6*c*/8 (Fig. S2). X_1_ leads to alternation of layers at *z* = 0, 2*c*/8, 4*c*/8, 6*c*/8, with only small or only large circles in each, but has no effect in the layers at *z* = *c*/8, 3*c*/8, 5*c*/8, 7*c*/8 (Fig. S3). The ordering scheme for Δ_5_ gives doubling of the *c*-repeat. The pattern has been laid out to show that it is the same as shown for the *P*2/*c* structure illustrated in Figure 2 of Yamauchi *et al.* (2009 ▸). By itself, it would conform to space group *Pbcm*.

The requirement that there are Fe with just two different formal charges (represented by circles of only two different sizes), present in equal proportions is not met by any single irrep, so at least two must operate in combination. One of the active irreps has to be Δ_5_ since it is the only one that gives doubling of the *c*-repeat with respect to the parent cubic structure. Combinations of Δ_5_ ordering in a 1:1 ratio with each of 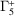, X_1_, and X_3_, *i.e.* Δ_5_ + 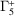, Δ_5_ + X_1_ and Δ_5_ + X_3_, give patterns of order which meet the requirement of equal proportions of two differently charged cations. The pattern shown in Fig. 1 ▸ is the ordering scheme of Yamauchi *et al.* (2009 ▸) which arises from a combination of Δ_5_ with X_1_. Layers at *z* = 0, 2*c*/8, 4*c*/8, 6*c*/8 alternate in having only one or other of the differently charged cations. Layers at *z* = *c*/8, 3*c*/8, 5*c*/8, 7*c*/8 each contain equal proportions of the two differently charged cations alternating in rows. Alternative patterns of order given by Δ_5_ + 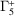 and Δ_5_ + X_3_ can be visualized by inspection using Figs. S2 + S1 and S2 + S4.

By itself the ordering scheme of Yamauchi *et al.* (2009 ▸), as illustrated in Fig. 1 ▸, would conform to space group *Pbcm*. This can be reached from the parent space group, *Fd*3*m*, by taking Δ_5_ as the symmetry of the active order parameter, *q*_Δ_, because a component *q*_X1_, with the symmetry of irrep X_1_, is then present as a secondary order parameter. The full output from *ISOTROPY* for this symmetry change is given in Table S2. Coupling between order parameter components with symmetries Δ_5_ and X_1_ would have the form 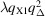 in lowest order but biquadratic coupling, 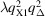, is always allowed. The requirement of a 1:1 ratio of Fe atoms with different formal charges on the octahedral sites appears to imply a rigid dependence of *q*_X1_ on *q*_Δ_. In the case of the 1:1 ordering resulting from Δ_5_ + X_3_ the same argument would apply to ensure a strict dependence of *q*_X3_ on *q*_Δ_. In other words, it is assumed that the favoured ordering scheme depends, effectively, on *q*_Δ_ alone.

Coupling between the order parameter with symmetry 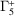, *q*_Γ_, and the order parameter with symmetry Δ_5_, *q*_Δ_, is linear/quadratic, 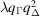. On this basis the generalized coupling behaviour for ‘‘electronic’’ and ‘‘cation charge order’’ contributions to the *P*2/*c* structure is λ*Q*_e_*Q*_co_^2^. Quotation marks have been added here to emphasize that the form of coupling depends on symmetry arguments rather than specifics of the atomic scale driving mechanisms. Here and throughout, we use upper case *Q* to represent the generic coupling without reference to symmetry and lower case *q* to represent order parameters or order parameter components with specific symmetry.

### *Cc* (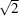
*a* × 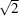
*a* × 2*a*)

2.2.

The full list of irreps for *Fd*3*m* → *Cc* includes eight with symmetry of the Γ point (0,0,0), three with symmetry of the Δ point (0,1/2,0), four with symmetry of the X point (0,1,0) and two with symmetry of the W point (1/2,1,0) (Table S3). Just as for *Fd*3*m* → *P*2/*c*, a combination of order parameters with the symmetries of at least two irreducible representations (irreps) of space group *Fd*3*m* is required to achieve the reduction in symmetry. The combinations listed in Table S3 automatically generate an order parameter with symmetry 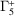. By symmetry, the transition has to be first order in character.

Different cation charge ordering schemes on the octahedral sites can be generated by irreps 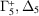, X_1_ and X_3_ with, in comparison with the *P*2/*c* structure, additional degrees of freedom implied by the increased number of independent components in each of Δ_5_, X_1_ and X_3_. Two further irreps, W_1_ and W_2_, are also permissive of ordering and there are two different patterns of ordering associated with each of these (Figs S5–S8). The two configurations of W1 differ by an offset of 1/4*c* at layers *z* = 0, 2*c*/8, 4*c*/8, 6*c*/8 with respect to layers at *z* = *c*/8, 3*c*/8, 5*c*/8, 7*c*/8 (compare Fig. S5 with Fig. S6). The same applies to the two configurations of W2 (Figs. S7 and S8).

Arrangements for ordering of equal proportions of Fe with two different formal charges on the octahedral sites still require the operation of Δ_5_ with some fixed combination of the other irreps. There are multiple ways in which ordering schemes of different irreps could be combined in attempts to reproduce the pattern of ordering shown in Figure 3 of Yamauchi *et al.* (2009 ▸) for the *Cc* structure, and no attempt has been made here to explore these in full. The key symmetry argument remains that the essential coupling term for the transition as a whole has the form 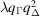.

## 3. Strain analysis

Spontaneous strains presented in symmetry-adapted form provide an indirect means of evaluating the evolution of different order parameters in systems with phase transitions that depend on multiple order parameters (McKnight *et al.*, 2009 ▸; Carpenter *et al.*, 2005 ▸; Carpenter & Howard, 2009*a* ▸, 2009*b* ▸, 2010*b* ▸; Eckstein *et al.*, 2022 ▸). In the case of the *P*2/*c* model structure of magnetite, there is a minimum of three order parameters to consider and these appear to have the symmetry of 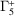, Δ_5_ and X_1_. Following the argument in the previous section in relation to the requirement that, for an equal proportion of only two Fe cations with different formal charges on the octahedral sites, it is assumed that there is only one independent order parameter for the charge order component of the transition. The single order parameter used here is *q*_Δ_, with the understanding that the pattern of ordering depends on some combination of Δ_5_ and X_1_ (and/or X_3_) ordering schemes in fixed proportions. The same argument applies to the *Cc* structure; *i.e.* it is assumed that X_1_, X_3_, W_1_ and W_2_ contributions to the charge ordering at octahedral sites do not vary independently with respect to *q*_Δ_.

The volume strain, *e*_a_ (= *e*_1_ + *e*_2_ + *e*_3_) has the symmetry of 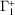. *e*_t_ [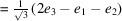] and *e*_o_ (= *e*_1_ − *e*_2_) are tetragonal and orthorhombic shear strains, respectively, and have the symmetry of 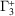. The remaining shear strains, *e*_6_, *e*_4_ and *e*_5_, have the symmetry of 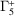. The 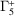 order parameter for the *P*2/*c* and *Cc* structures has three components, *q*_Γ1_ ≠ *q*_Γ2_ = *q*_Γ3_. The Δ_5_ order parameter has two components, *q*_Δ1_ ≠ *q*_Δ2_ ≠ 0 for *Cc* as the subgroup symmetry and one, *q*_Δ1_ ≠ 0, *q*_Δ2_ = 0, for subgroup *P*2/*c*. When treated as driving two separate but coupled instabilities, the temperature dependence of these two order parameters and their coupling with strain would be expected to conform to solutions of a Landau expansion with the form:
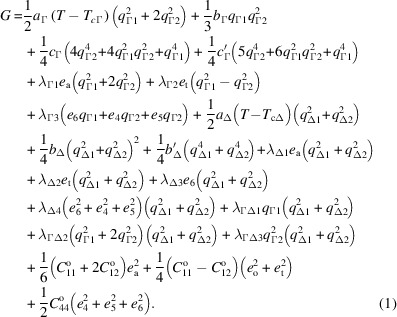
Here *a*, *b*, *c* are standard coefficients with subscripts to identify which order parameter they refer to. *T*_cΓ_ and *T*_cΔ_ represent critical temperatures for the two instabilities. λ_Γ1_, λ_Γ2_, *etc*. are coupling coefficients and 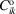, *i*, *k* = 1–6, represent bare elastic moduli, *i.e.* excluding the influence of the phase transition. The lowest order term for direct coupling between the two order parameters is linear/quadratic, 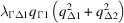, but there are also biquadratic terms, 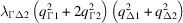 and 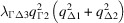. The *P*2/*c* and *Cc* structures both have *e*_a_ ≠ 0,*e*_t_ ≠ 0,*e*_o_ = 0,*e*_6_ ≠ *e*_4_ = *e*_5_.

Setting the equilibrium conditions 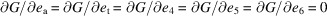 gives
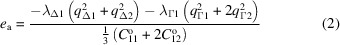

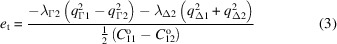

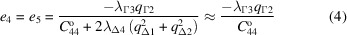

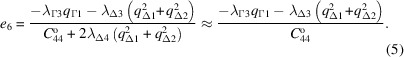


**Figure 2:**
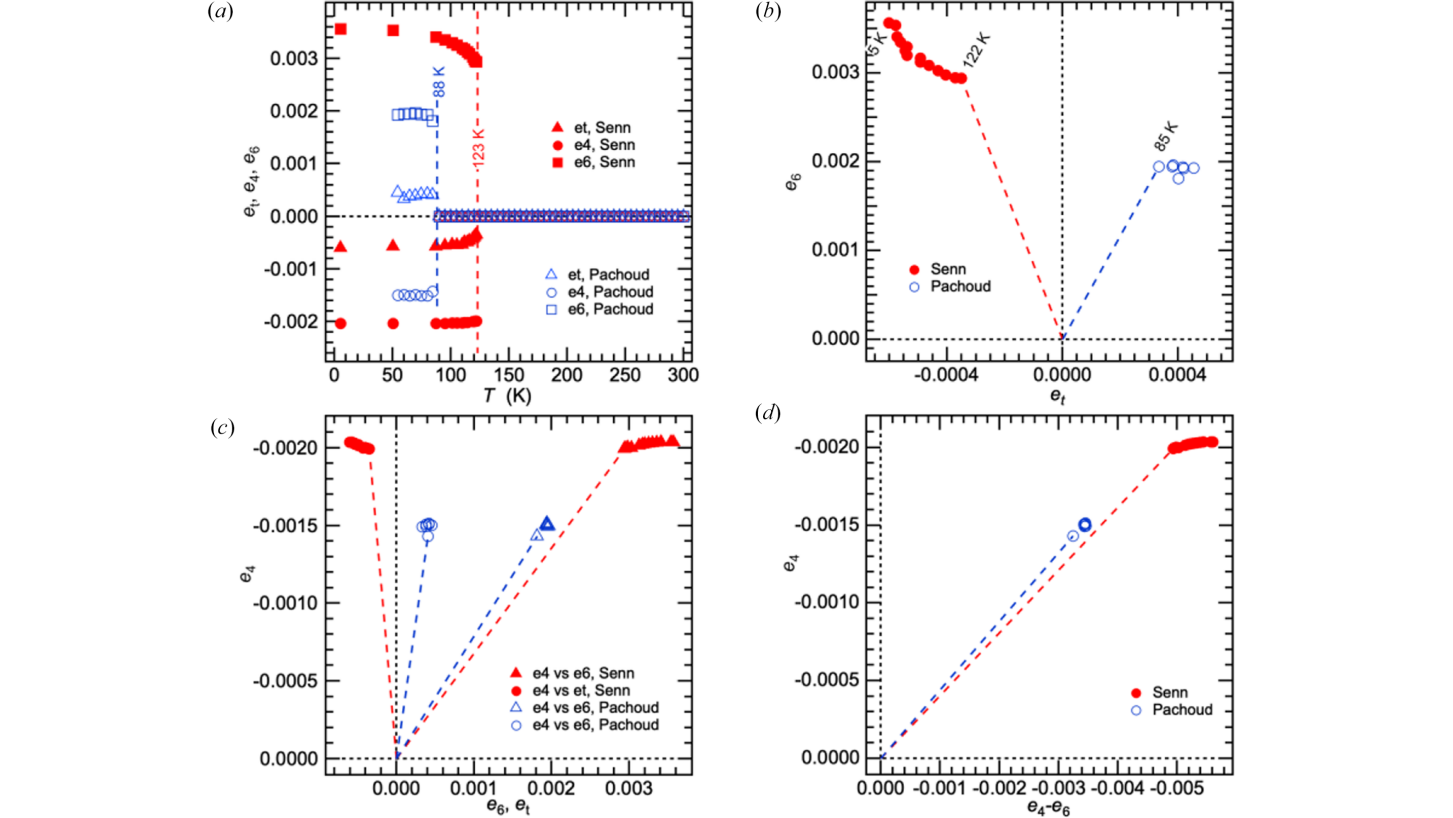
Variations of shear strains determined from lattice parameters given in Figure 3 of Senn *et al.* (2015 ▸) for a synthetic magnetite sample with *T*_v_ ∼123 K, and in Figure 1 of Pachoud *et al.* (2020 ▸) for a synthetic sample with estimated composition *x* = 0.0228 in Fe_3–*x*_Zn*_x_*O_4_. Pachoud *et al.* quoted *T*_v_ = 92 K for their sample but the discontinuity in lattice parameters occurred between 85 and 90 K in their lattice parameter data. Original lattice parameters and expressions for calculation of the strains are given in Fig. S10 and Table S4, respectively, with respect to the reference system shown in Fig. S9. Coloured dashed lines in (*b*), (*c*), (*d*) represent discontinuities at *T* ∼ *T*_v_.

Fig. 2(*a*) ▸ includes values for the shear strains extracted from lattice parameter data given in Figure 3 of Senn *et al.* (2015 ▸), using expressions listed in Table S4 for the crystallographic orientation shown in Fig. S9. Values of the original lattice parameters are shown in Fig. S10. *e*_4_ is expected to scale with *q*_Γ2_ [equation (4)] and displays the strongly first order character of the transition. *e*_t_ and *e*_6_ both arise in part by coupling with 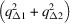 [equations (3) and (5)] and show a less abrupt temperature dependence as *T*_v_ is approached from below.

If there was only one intrinsic instability, the three shear strains would evolve in a more closely similar manner because the order parameters representing electronic and charge order contributions would vary in fixed proportions with each other. In particular, if the transition depended only on 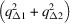, *e*_6_ would be a linear function of *e*_t_, passing through the origin. Fig. 2 ▸(*b*) shows that this dependency is not observed, consistent with a formulation in terms of coupling between two separate instabilities. Variations of *e*_4_ with respect to *e*_6_ and *e*_t_ are shown in Fig. 2 ▸(*c*) for completeness.

By itself, an electronic order parameter with *q*_Γ1_ = *q*_Γ2_ = *q*_Γ3_ would produce a rhombohedral strain, *e*_4_ = *e*_5_ = *e*_6_, with *e_i_* ∝ *q_i_*. Ordering on the basis of the Δ_5_ order parameter with *q*_Δ1_ ≠ 0, *q*_Δ2_ = 0 alone would yield a structure in space group *Pbcm*. Addition of the orthorhombic strains to the rhombohedral strains produces the monoclinic distortion, *e*_6_ ≠ *e*_4_, in space group *P*2/*c* and is accompanied by relaxations attributable to the difference between *q*_Γ1_ and *q*_Γ2_. Ordering on the basis of the Δ_5_ order parameter with *q*_Δ1_ ≠ *q*_Δ2_ ≠ 0 alone would also yield an orthorhombic structure, this time in space group *Pmc*2_1_. The combination of orthorhombic and rhombohedral strains would then give the same monoclinic distortion, but in space group *Cc*.

The relationship between *e*_4_ (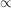
*q*_Γ2_) and *e*_4_ − *e*_6_ {≃ [λ_Γ3_(*q*_Γ1_ − *q*_Γ2_) + λ_Δ3_(*q*_Δ1_^2^ + *q*_Δ2_^2^)](*C*_44_^o^)^−1^} in Fig. 2 ▸(*d*), shows that *e*_4_ remains nearly constant in comparison with the contributions from relaxations due to (*q*_Γ1_ − *q*_Γ2_) and 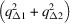 within the stability field of the monoclinic structure.

Determination of values for the volume strain, *e*_a_, would require sufficient lattice parameter data in the stability field of the high-symmetry phase to allow extrapolation of a baseline into the stability field of the low-temperature phase. In the absence of data for the cubic structure of magnetite above *T*_v_, it has not been possible to determine values of *e*_a_ for the synthetic magnetite sample of Senn *et al.* (2015 ▸) (*T*_v_ ∼123 K). However, data reported for a wider temperature interval by Wright *et al.* (2000 ▸), and reproduced in Fig. S11, show that the transition is accompanied by a small increase in volume for a synthetic sample with *T*_v_ ∼ 108 K. A similar positive volume strain is evident in the lattice parameter data shown in Fig. 6 of Kozlowski *et al.* (1999 ▸) for a synthetic crystal with *T*_v_ ∼ 120 K.

**Figure 3:**
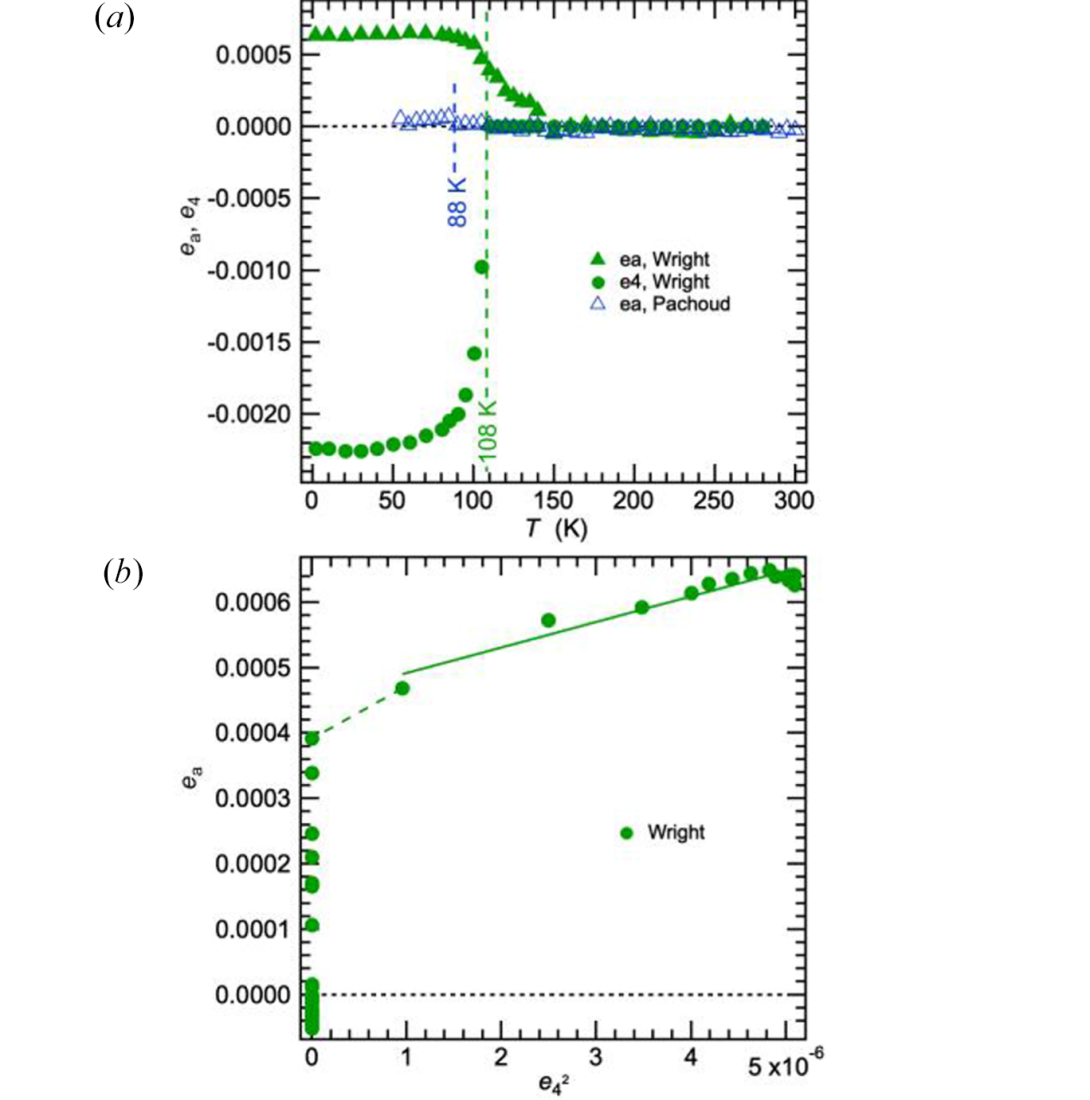
Variations of spontaneous strains determined from lattice parameters given in Figure 3 of Wright *et al.* (2000 ▸) for a synthetic magnetite sample with *T*_v_ = 108 K. The original lattice parameters are reproduced in Fig. S11 and expressions for the strains are given in Table S4. Also included in (*a*) is the volume strain, *e*_a_, determined from lattice parameter data of Pachoud *et al.* (2020 ▸) for a synthetic magnetite crystal with estimated composition *x* = 0.0228 in Fe_3–*x*_Zn*_x_*O_4_. (Pachoud *et al.* (2020 ▸) reported *T*_v_ = 92 K for this sample; the small discontinuity in *e*_a_ deternined from their data is shown here as being at 88 K). The dashed line in (*b*) represents the first order discontinuity at ∼*T*_v_. The solid line is a fit to data at *T* < *T*_v_.

Lattice parameters of Wright *et al.* for the low temperature structure were refined under rhombohedral symmetry (*e*_4_ = *e*_5_ = *e*_6_). Values of the strains *e*_a_ and *e*_4_ obtained from these are shown in Fig. 3 ▸(*a*) using expressions given in Table S4. Although the magnitude of *e*_4_ at low temperatures is closely similar to that shown in Fig. 2 ▸(*a*), it has a steeper temperature dependence as *T*_v_ is approached from below. If the transition was driven only by the electronic component, as represented by *q*_Γ1_ (= *q*_Γ2_ = *q*_Γ3_), the two non-zero strains would be expected to vary as *e*_a_ ∝ *e*_4_^2^ ∝ *q*_Γ1_^2^ [equations (2) and (4)]. As seen in Fig. 3 ▸(*b*), *e*_a_ and *e*_4_^2^ display a linear correlation but the straight line fit to the data does not pass through the origin. The simplest explanation is that there are two contributions to the volume strain [equation (2)] and that deviations from ideal stoichiometry cause shear strain coupling with long range charge order to be suppressed. Non-zero values of *e*_a_ between *T*_v_ and ∼150 K are indicative of precursor effects arising from one or both of the electronic and charge order contributions. For comparison with data for the crystal with *T*_v_ = 123 K, spontaneous strain values determined from lattice parameter data given in Fig. 1 ▸(*c*) of Pachoud *et al.* (2020 ▸) have been added to Fig. 2 ▸. The sample of Pachoud *et al.*, Fe_3–*x*_Zn*_x_*O_4_, was described as having *T*_v_ = 92 K and estimated composition *x* = 0.0228. The original lattice parameters from refinement in space group *Cc* are included in Fig. S10. All the shear strains have values which are lower than for the pure magnetite sample of Senn *et al.* (2015 ▸) but, apart from a change in sign for values of *e*_t_, the overall pattern of their temperature dependence is similar. Pachoud *et al.* reported data for the cubic lattice parameter between *T*_v_ and 300 K (Fig. S10), from which values of *e*_a_ have been determined using values of *a*_o_ extrapolated into the stability field of the monoclinic phase. As shown in Fig. 3 ▸(*a*), they are not distinguishable from zero.

Heat capacity measurements have shown that increased doping leads to a change from first order character towards second order character (Kozłowski *et al.*, 1996 ▸, 1997 ▸, 2000 ▸). The data in Fig. 2 ▸ for the sample with *T*_v_ ∼ 88 K show a distinct first order step in the shear strain rather than a tendency to become more nearly second order, however. This could be an issue of experimental resolution arising from the difficulty in following changes in lattice parameters when distortions from cubic geometry become very small.

## 4. Symmetry-adapted atomic displacements

One means of characterizing the evolution of different components of a symmetry change involving as many possible secondary irreps as occurs in the present case is to follow the pattern of symmetry-adapted atomic displacement distances from structure refinements. Advantage is taken here, therefore, of values of symmetry-adapted displacement modes reported by Senn *et al.* (2015 ▸) for the refined *Cc* structure, with respect to the high symmetry (*Fd*3*m*) structure. *ISODISTORT* from the *ISOTROPY* software suite had been used to follow a total of 168 modes. While the amplitudes of these do not provide direct information on the pattern of cation charge ordering, the temperature dependence of displacement modes with symmetry 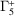, Δ_5_, X_1_, X_3_, W_1_ and W_2_ will be indicative also of the temperature dependence of ordering on the basis of the same symmetry elements.

**Figure 4:**
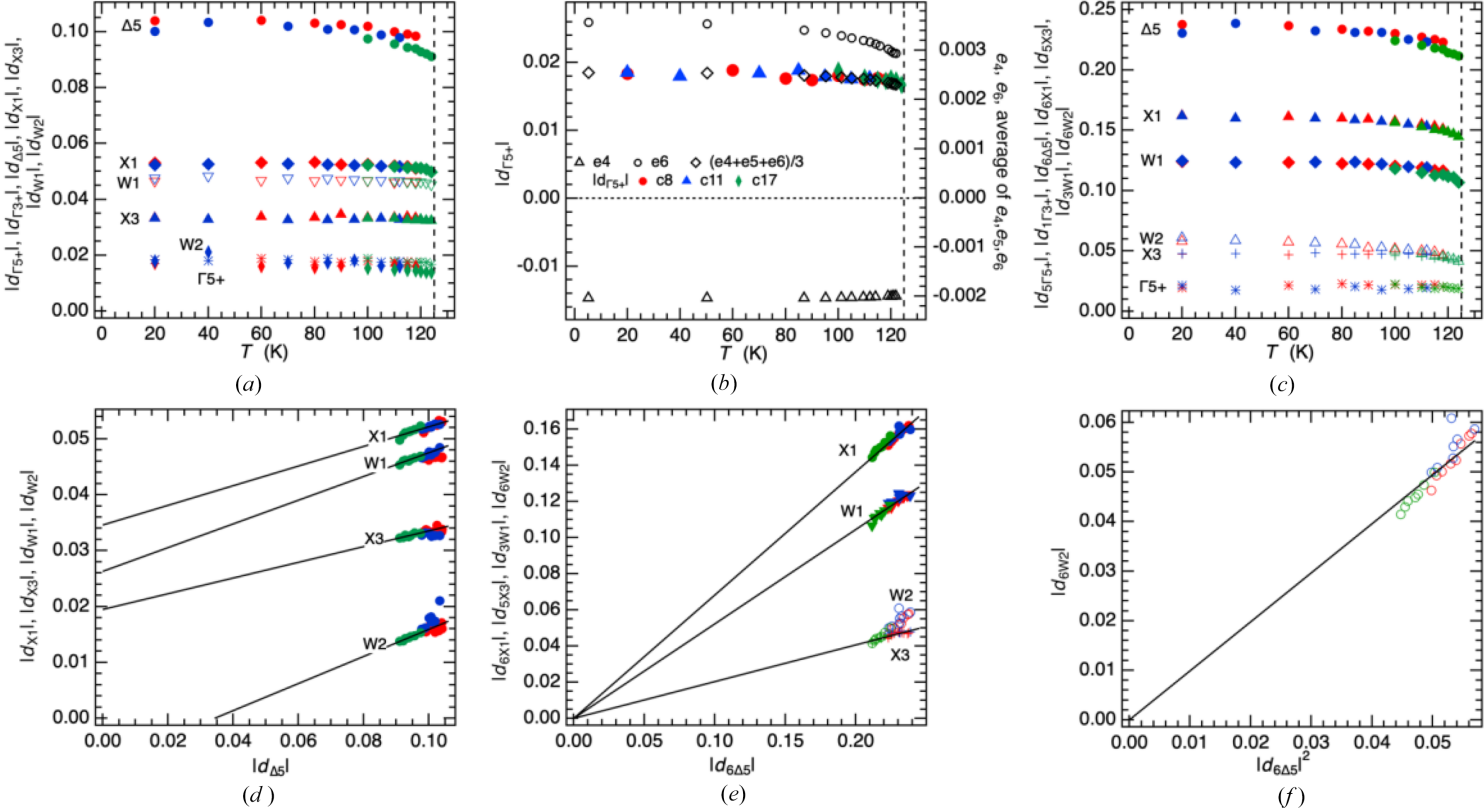
Variation of symmetry-adapted atomic displacements, |*d*|, with respect to the parent cubic structure, derived from original data of Senn *et al.* (2015 ▸) refined in space group *Cc*. Three different crystals, c8 (red), *c*11 (blue), *c*17 (green), were from the same original batch as used for determination of the temperature dependence of lattice parameters shown in Fig. S10a. (*a*) Temperature dependence of average values of displacements with symmetry 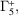 Δ_5_, X_1_, X_3_, W_1_ and W_2_. (*b*) Comparison of the average displacement amplitude |*d*_Γ5+_| with variations of *e*_4_ and *e*_6_, showing that it has the same temperature dependence as the average value of shear strains belonging to irrep 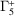. (*c*) Temperature dependence of values of individual displacement modes with symmetry 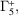 Δ_5_, X_1_, X_3_, W_1_ and W_2_. 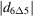 is mode 37 in the list of modes from Senn *et al.* (2015 ▸), 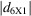 is mode 43, 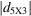 is mode 78, 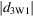 is mode 61, 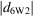 is mode 68. (*d*) Variations of average |*d*| values for different symmetries with respect to variation of |*d*_Δ5_|: all are approximately linear but straight lines through the data do not pass through the origin. (*e*) Comparison of individual |*d*| variations selected as displaying the largest temperature dependences for different symmetries with the variation of the individual values 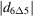: straight lines through the data were constrained to pass through the origin showing that the X_1_, W_1_ and X_3_ modes can be understood as scaling linearly with the Δ_5_ mode. (*f*) A straight line through data, constrained to pass through zero, shows that the W_2_ mode can be understood as scaling with the square of the Δ_5_ mode.

In the case of 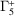, there are six symmetry-adapted displacements, *d_i_* (*i* = 1–6), and the average of their amplitudes is |*d*_Γ5+_| = (|*d*_1_| + |*d*_2_| + |*d*_3_|…)/6. The same treatment for |*d*_Δ5_| (22 modes), |*d*_X1_| (28 modes), |*d*_X3_| (15 modes), |*d*_W1_| (20 modes), and |*d*_W2_| (22 modes) yielded the values shown in Fig. 4 ▸(*a*). All show essentially the same pattern of a discontinuity at *T*_v_ followed by a small increase before levelling off below ∼80 K. Each irrep contains multiple components and, in principle, the average displacement |*d*_Γ5+_| should show the same temperature dependence as the average 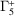 shear strain, (*e*_4_+*e*_5_+*e*_6_)/3. The numbers are small but the data in Fig. 4 ▸(*b*) are consistent with this. However, individual sets of values of *d_i_* show variations in their temperature dependences from flat to significantly curved, with the implication that using average values hides potential information about the variations of individual modes belonging to each irrep. It has already been shown by the evolution of *e*_4_ (∝ ∼*q*_Γ2_) in Fig. 2 ▸(*a*) that the electronic aspect of the order parameter shows very little temperature dependence, so the relatively steep temperature dependences can be ascribed predominantly to the cation charge ordering process. The values of *d_i_* shown in Fig. 4 ▸(*c*) were picked out as displaying the steepest temperature dependence and, hence, as potentially most revealing of order parameter components that relate to the ordering.

In their simplest form, allowed terms for coupling of ordering on the basis of irrep X_1_ with ordering on the basis of irrep Δ_5_ are 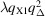 and 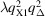. If the lowest order coupling term is dominant, a scaling *q*_X1_ ∝ 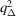 would be expected. If the cation charge ordering on the octahedral sites is rigidly constrained by the 1:1 ratio of Fe^2+^ to Fe^3+^, a fixed dependence from the higher order term as *q*_X1_ ∝ *q*_Δ_ is more likely. Fig. 4 ▸(*d*) demonstrates that average values of |*d_i_*| with different symmetries each vary approximately linearly with |*d*_Δ5_| but straight lines through them do not pass through the origin. A plot of |*d_i_*| averages against |*d*_Γ5+_|^2^ (not shown) gives essentially the same result - straight lines that do not pass through the origin. On the other hand, data from single modes showing the maximum temperature dependence plotted in the same way [Fig. 4 ▸(*e*)] show linear dependences that, for X_1_, W_1_ and X_3_ values, include the origin. The variation of values for the W_2_ mode are permissive of a W_2_ component scaling with values of the square of the Δ_5_ mode [Fig. 4 ▸(*f*)]. The numbers are small and spread over only narrow ranges, but Fig. 4 ▸(*e*), at least, is permissive of the rigid dependence of X_1_ ordering on Δ_5_ ordering assumed when developing the scheme shown in Fig. 1 ▸.

**Figure 5:**
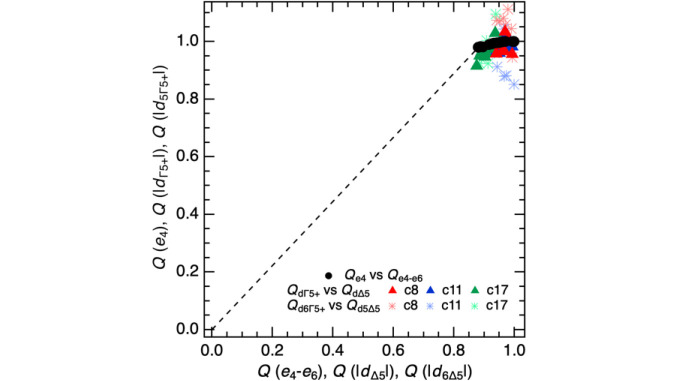
Proxies for the variation of order parameters with symmetry 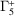 and Δ_5_, each scaled to *Q* = 1 at 0 K. The average and individual symmetry-adapted displacements do not depend on the same order parameter components as the shear strains but all three sets of data show the same pattern of evolution: a large discontinuity in the electronic and cation charge order components of the transition at *T*_v_ (broken line) is followed by small variations predominantly in the cation charge order component with further reduction in temperature.

Notwithstanding the assumptions involved in interpreting the symmetry-adapted displacements, Fig. 5 ▸ reveals a consistent pattern of evolution for the two principal order parameters referred to previously in a generic manner as 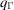 and *q*_Δ_. For this, the average values |*d*_Γ5+_| with |*d*_Δ5_| and values for single modes with the same symmetry were scaled so that they would each extrapolate to a value of *Q* = 1 at 0 K. A similar scaling of values of *e*_4_ and *e*_4_-*e*_6_ from Fig. 2 ▸(*d*) has been added. Scatter is greatest for the individual displacement modes and least for the strain variations, reflecting the relative degrees of experimental uncertainties. The shear strains and displacement amplitudes do not depend on exactly the same combinations of order parameter components but they show the same pattern of a discontinuity at *T*_v_ from zero to ∼0.85 for 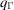 and ∼0.95 for *q*_Δ_. Below *T*_v_, 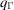 varies only slightly in comparison with a wider variation of *q*_Δ_, in a non-linear manner that is consistent with the evolution of two driving order parameters that are coupled but have separate critical temperatures.

## 5. Discussion

### 5.1. Linear/quadratic coupling for two instabilities with different critical temperatures

Consideration of the Verwey transition in magnetite from the perspective of multiple order parameters brings into focus coupling according to 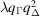. Although this does not require that the microscopic mechanisms are described, *q*_Γ_ has been discussed in terms of a (zone centre) electronic instability and *q*_Δ_ in terms of (zone boundary) ordering of Fe^2+^ and Fe^3+^ on octahedral sites. Coupling between the two order parameters could be direct, as expressed through the term 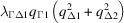, or indirect through the common strain, *e*_6_, due to the terms 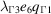 and 
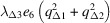
.

Following the generic treatment of Salje & Carpenter (2011 ▸) (and see, also, Cano *et al.*, 2010 ▸), linear/quadratic coupling 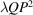 between order parameters *Q* and *P* can lead to two substantially different outcomes depending on the relative values of the critical temperatures, *T*_cP_ and *T*_cQ_. For *T*_cQ_ > *T*_cP_, the expectation would be for two discrete transitions and a sequence of structures with falling temperature as *Q* = *P* = 0 → *Q* ≠ 0, *P* = 0 → *Q* ≠ 0, *P* ≠ 0. For *T*_cP_ > *T*_cQ_ only one transition would be expected, *Q* = *P* = 0 → *Q* ≠ 0, *P* ≠ 0, with *Q* and *P* displaying different dependences on temperature through the stability field of the low-symmetry structure. It has been shown here that experimental data for symmetry-adapted strains and atomic displacements which scale with the two proposed order parameters in magnetite are consistent with different temperature dependences below a single, discrete transition at *T*_v_. Details of the Landau expansion [equation (1)] are more complicated than the generic expression used by Salje & Carpenter (2011 ▸), but the implication is that *T*_cΔ_ is most likely greater than *T*_cΓ_.

### 5.2. Chemical doping and local strain heterogeneity

Changes in oxidation state balanced by increasing vacancy concentrations on the cation sites and substitution of Zn, Ti or Al for Fe by up to ∼3.5% causes *T*_v_ to reduce from ∼125 K to ∼80 K (Kozłowski *et al.*, 1996 ▸, 1997 ▸, 1999 ▸, 2000 ▸; Kołodziej *et al.*, 2012 ▸; Goto & Lüthi, 2003 ▸; Kąkol *et al.*, 2012 ▸). However, nonlinear softening of *C*_44_ as *T* → *T*_v_ from above, diagnostic of a pseudoproper ferroelastic transition, is unaffected by doping with Zn (Fig. 6 of Schwenk *et al.*, 2000 ▸). The softening is described by *C*_44_ = 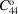 (*T* − 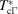)/(*T* − *T*_cΓ_), where 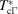 is the value of *T*_cΓ_ renormalized by bilinear coupling of *q*_Γ2_ with *e*_4_ (at *q*_Δ1_ = *q*_Δ2_ = 0) and 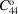 is the value of *C*_44_ in the absence of the instability.

Schwenk *et al.* (2000 ▸) obtained the same values of 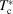 and 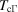, 66 K and 56 K, respectively, for three different compositions, *x* = 0, 0.02, 0.032 in Fe_3–*x*_Zn*_x_*O_4_, implying that the Γ-point instability is not suppressed by doping and that the strength of bilinear coupling, as represented by the value of λ_Γ3_ in equation (1), is also unaffected. The dominant influence of changes in composition at this level appears to be related predominantly to the cation charge order component of the phase transition, therefore. Given that almost identical lowering of *T*_v_ is seen as a function of composition for different substituting cations (Figure 2 of Kąkol *et al.* 2012 ▸), the effect is more likely to have an effectively physical rather than purely chemical origin. A simple explanation in this context relates to local strain heterogeneity accompanying the substitution of small spheres for large spheres, or vice versa, in a more or less elastic matrix.

In the case of silicate solid solutions, hard mode infrared spectroscopy has shown that cations of different sizes are accommodated by the development of local strain heterogeneity on a length scale of a few unit cells (Atkinson *et al.*, 1999 ▸, 2024 ▸; Boffa Ballaran *et al.*, 1998 ▸; Carpenter *et al.*, 1999 ▸; Carpenter & Boffa Ballaran, 2001 ▸). The influence of such local strain heterogeneities on phase transitions is seen most clearly in the plateau effect, whereby the transition temperature for a displacive transition in a pure crystal is unaffected by chemical substitutions at the lowest concentrations. The temperature of a thermodynamically continuous transition only starts to change once some critical doping level has been reached, corresponding to the point at which strain fields round individual replacement cations start to overlap. For example, the plateau of nearly constant temperature for the displacive transition in NaAlSi_3_O_8_ extends to ∼2% substitution of K^+^ (∼1.5 Å) for Na^+^ (∼1.0 Å) implying that the strain fields around individual K^+^ ions have diameters of ∼20–40 Å (Carpenter *et al.*, 1999 ▸). In the case of La^3+^ (∼1.03 Å) substitution for Pr^3+^ (∼0.99 Å) in the perovskite PrAlO_3_, the plateau for a transition at ∼150 K extends to 1.6 ± 0.2%, implying that individual strain fields around La^3+^ have a diameter of ∼16–18 Å (Carpenter *et al.*, 2009 ▸).

Strain heterogeneities must exist in doped magnetite but *T*_v_ for the Verwey transition does not show a discrete plateau with increased doping. This could be due to overlapping of strain fields at smaller doping levels in a close packed oxide structure, and/or to the complication of two order parameters interacting at a first order transition. Nevertheless, there is a break in the trend of decreasing *T*_v_ with composition at ∼1.3% substitution of Fe by Zn, Ti, Al or vacancies [as summarized in Figure 4 of Attfield (2022 ▸)], which correlates with the plateau limit in other materials and hints at some analogous influence of local strain fields on the transition.

### 5.3. Local strain heterogeneity and suppression of macroscopic strain

An additional consequence of local strain heterogeneity in systems with cation ordering is that coherent macroscopic strains become suppressed by the introduction of site disorder. For example, crystals of the perovskite La_0.6_Sr_0.1_TiO_6_ can be prepared with either ordered or disordered distributions of vacancies on the *A*-cation site. The disorder is accommodated, in part at least, by the development of local strain heterogeneity that is eliminated when the vacancies evolve to an ordered configuration. The same displacive phase transition occurs in samples with disordered vacancies as when they are ordered but the macroscopic spontaneous strain is almost entirely suppressed when the crystals are prepared with a disordered state (Howard *et al.*, 2007 ▸). Comparison in Fig. 2 ▸(*a*) of results for the undoped crystal (*T*_v_ = 123 K) and the Zn-doped crystal (*T*_v_ = 92 K) shows that, in addition to lowering *T*_v_, the effect of Zn-doping is to reduce the magnitude of the shear strains, consistent with the experience from perovskites. The volume strain is suppressed to essentially zero [Fig. 3 ▸(*a*)].

Evidence from the evolution of *C*_44_ at *T* > *T*_v_ indicates that bilinear coupling 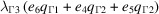 is not affected significantly by changes in composition, implying that the suppression of shear strain *e*_6_ by the introduction of extraneous cations or vacancies amounts primarily to lowering of values of the coupling coefficient λ_Δ3_ in the term 
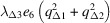
. If 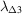 is reduced, the contribution of coupling via the common strain *e*_6_ to the effective linear/quadratic coupling between *q*_Γ_ and *q*_Δ_ will also be reduced. This in turn is likely to account for suppression of the transition, both in terms of lowering values of *T*_v_ and reducing the magnitude of *q*_Γ1_.

Suppression of the volume strain by doping [Fig. 3 ▸(*a*)] implies that either or both of the coupling coefficients in 
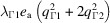
 and 
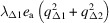
 is/are reduced. A standard result of order parameter coupling with volume strain, as in the case of the α–β transition in quartz (Carpenter *et al.*, 1998 ▸), is that the fourth order coefficient in a Landau expansion is renormalized such that strong coupling drives the transition towards first order character. Suppression of this coupling in magnetite must be a contributory factor to the change from first order character towards second-order character with increased doping seen in the heat capacity measurements of Kozłowski *et al.* (1996 ▸, 1997 ▸, 2000 ▸). By symmetry the transition is first order in character due to the presence of the third-order term, 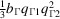. However, *b*_Γ_ is a property of the material and can be small, as appears to be the case if the transition becomes close to second order rather than simply being smeared over some temperature interval in doped samples.

The third significant difference in strain evolution between the pure and doped samples is the change in sign of *e*_t_ [Fig. 2 ▸(*a*)]. According to equation (3), the implication is that λ_Γ2_ and λ_Δ2_ in 
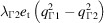
 and 
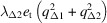
 have opposite sign. Reduction in the value of one of the coefficients would change the sign of the sum of strain contributions to the overall value of *e*_t_, as would differential reduction in the values of 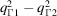 and 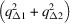. If this is the case, biquadratic coupling of the two order parameters via *e*_t_ as a common strain would be unfavourable, contributing to rather complicated patterns of evolution of the order parameters below *T*_v_ with and without doping.

### 5.4. *Cc* structure

The structure reported by Yamauchi *et al.* (2009 ▸) in space group *P*2/*c* indicated that, if the cation charge ordering depended only on distributing large Fe^2+^ ions and small Fe^3+^ ions on octahedral sites in a ratio of 1:1, the preferred pattern by itself would have symmetry *Pbcm*. In this case the Δ_5_ irrep has only one component and the ordering is permitted by irreps Δ_5_ and X_1_ in fixed proportions. The pattern obtained from their calculations in space group *Cc* has differences due, presumably, to stabilization achieved by clustering to form the trimerons identified by Senn *et al.* (2012*a* ▸). Symmetry-adapted atomic displacement amplitudes are permissive of linear dependence of ordering on the basis of X_1_ symmetry with ordering on the basis of Δ_5_. They also provide some indication of which additional symmetry components should be investigated as being most significant in this context. The overall picture is of the discontinuity at *T*_v_ being to a well ordered structure with only small increases in the degree of order with further falling temperature.

## 6. Conclusion

Equation (1) provides a practical description for the Verwey transition using the minimum number of independent order parameters required to give the observed symmetry change. It serves to emphasize that the transition belongs to an important class of phase transitions in multiferroic materials where linear/quadratic coupling between two order parameters defines the form of interaction between separate instabilities. As with other examples of linear/quadratic coupling referred to in *Introduction*[Sec sec1], the characteristic features are multiple order parameters, strong coupling via common strains, diverse patterns of elastic constant variations and diverse patterns of behaviour with changing composition, depending on how the critical temperatures of the two instabilities vary.

As in previously described examples, the zone centre instability is electronic. In general, there are many possibilities for the zone boundary instability, including magnetism and structural changes such as octahedral tilting in perovskites. In magnetite both order parameters are related to changes in electronic structure in the sense that the cation charge order arises from the cooperative Jahn–Teller instability of Fe^2+^. As with phase transitions in all these materials, the role of strain at both local and macroscopic length scales is fundamental in controlling the overall structural evolution and microstructure.

## 7. Related literature

The following references are cited in the supporting information: Carpenter (2007 ▸); Meyer *et al.* (2000 ▸); Meyer *et al.* (2001 ▸); Salje *et al.* (1991 ▸); Sondergeld *et al.* (2000 ▸).

## Supplementary Material

Tables S1-S4, Figs. S1-S11. DOI: 10.1107/S2052520625004779/dk5138sup1.pdf
