# Supplementary material for: A linear/quadratic order parameter coupling description of the Verwey transition in magnetite, Fe3O4
Source: Acta Crystallogr B Struct Sci Cryst Eng Mater. 2025 Jul 8;81(Pt 4):427–36. doi: 10.1107/S2052520625004779 (PMC12322932; doi:10.1107/S2052520625004779)
Supplement: Supplementary file 1 [file b-81-00427-sup1.pdf]

STRUCTURAL SCIENCE
CRYSTAL ENGINEERING
MATERIALS

Volume 81 (2025)

Supporting information for article:

A linear/quadratic order parameter coupling description of the Verwey transition in magnetite, Fe3O4

Michael A. Carpenter, Richard J. Harrison, James Shaw-Stewart, Kanta Adachi, Mark S. Senn and Christopher J. Howard

Table S1. Active irreps and order parameters given by ISOTROPY for the transition $Fd\bar{3}m \rightarrow P2/c$, with basis (1/2,1/2,0), (-1/2,1/2,0), (0,0,2) and origin (0,0,0). The table also shows subgroups that would result if each of these irreps operated alone. It is found using ISODISTORT that at least two active irreps are required to achieve the change in symmetry. Possible pair combinations are listed; none can lead to a transition which is allowed to be thermodynamically continuous.

Irrep (ML)	k params	k vector	Dir	Subgroup	Size
GM1+		(0,0,0)	(a)	227 Fd-3m	1
GM3+		(0,0,0)	(a,0)	141 I4_1/amd	1
GM4+		(0,0,0)	(a,-a,0)	12 C2/m	1
GM5+		(0,0,0)	(a,b,b)	12 C2/m	1
DT2	1/4	(0,1/2,0)	(0,0,0,0,a,0)	54 Pcca	4
DT4	1/4	(0,1/2,0)	(0,0,0,0,0,a)	54 Pcca	4
DT5	1/4	(0,1/2,0)	(0,0,0,0,0,0,0,0,0,a,a,0)	57 Pbcm	4
X1		(0,1,0)	(0,0,0,0,a,0)	51 Pmma	2
X3		(0,1,0)	(0,0,0,0,a,-a)	53 Pmna	2

Possible sets of primary order parameters (from ISODISTORT):

GM4+ & DT2, GM5+ & DT2, GM4+ & DT4, GM5+ & DT4, GM4+ & DT5, GM5+.& DT5, DT2 & DT5, DT4 & DT5, DT2 & X3, DT4 & X3, DT5 & X3.

Table S2. Acitve irreps and order parameters given by ISOTROPY for the transition $Fd\bar{3}m \rightarrow Pbcm$, with basis (-1/2,1/2,0), (0,0,2), (1/2,1/2,0) and origin (0,0,0). The table also shows subgroups that would result if each of these irreps operated alone. The transition is not allowed to be thermodynamically continuous.

Irrep (ML)	k params	k vector	Dir	Subgroup	Size
GM1+		(0,0,0)	(a)	227 Fd-3m	1
GM3+		(0,0,0)	(a,0)	141 I4_1/amd	1
GM5+		(0,0,0)	(a,0,0)	74 Imma	1
DT5	1/4	(0,1/2,0)	(0,0,0,0,0,0,0,0,0,a,a,0)	57 Pbcm	4
X1		(0,1,0)	(0,0,0,0,a,0)	51 Pmma	2

Table S3. Active irreps and order parameters given by ISODISTORT for the transition $Fd\bar{3}m \rightarrow Cc$, with basis (1,1,0), (-1,1,0), (0,0,2) and origin (0,0,1/2). At least two active irreps are required to achieve this change in symmetry. Possible pair combinations are listed; none can lead to a transition which is allowed to be thermodynamically continuous.

Irrep (ML)	k params	k vector	Dir	Subgroup	Size
GM1+		(0,0,0)	(a)	227 Fd-3m	1
GM3+		(0,0,0)	(a,0)	141 I4_1/amd	1
GM4+		(0,0,0)	(a,-a,0)	12 C2/m	1
GM5+		(0,0,0)	(a,b,b)	12 C2/m	1
GM2-		(0,0,0)	(a)	216 F-43m	1
GM3-		(0,0,0)	(a,0)	119 I-4m2	1
GM4-		(0,0,0)	(a,a,b)	8 Cm	1
GM5-		(0,0,0)	(0,a,-a)	46 Ima2	1
DT2	1/4	(0,1/2,0)	(0,0,0,0,a,b)	27 Pcc2	4
DT4	1/4	(0,1/2,0)	(0,0,0,0,a,b)	27 Pcc2	4
DT5	1/4	(0,1/2,0)	(0,0,0,0,0,0,0,0,a,b,b,-a)	26 Pmc2_1	4
X1		(0,1,0)	(a,b,a,b,c,d)	8 Cm	4
X2		(0,1,0)	(a,b,-a,-b,0,0)	8 Cm	4
X3		(0,1,0)	(a,b,-b,-a,c,-c)	8 Cm	4
X4		(0,1,0)	(a,b,b,a,c,c)	8 Cm	4
W1		(1/2,1,0)	(0,0,0,0,a,b,-a,b,0,0,0,0)	8 Cm	4
W2		(1/2,1,0)	(0,0,0,0,a,b,a,-b,0,0,0,0)	8 Cm	4

Possible sets of primary order parameters (from ISODISTORT):

DT2.& X1, DT4 & X1, DT5 & X1, DT2 & X2, DT4 & X2, DT5 & X2, DT2 & X3, DT4 & X3, DT5 & X3, DT2 & X4, DT4 & X4, DT5 & X4, DT2 & W1, DT4 & W1, DT5 & W1, X1 & W1, X2 & W1, X3 & W1, X4 & W1, DT2 & W2, DT4 & W2, DT5 & W2.

Table S4. Expressions used to determine values of spontaneous strain components e_i, i = 1-6, from lattice parameters a, b, c, β of the *Cc* structure using data from Senn *et al*. (2015) (left) and a, α from refinements with rhombohedral lattice geometry in Figure 3 of Wright *et al*. (2000). a_o is the lattice parameter of the parent structure extrapolated into the stability field of the low temperature structure. For determinations of strain values using the data of Senn *et al*., values of a_o were estimated as $a_o = \left(\frac{abc\sin\beta}{4}\right)^{1/3}$. For determinations of strain values using the data of Wright *et al*., values of a_o were determined by first fitting data for the a-parameter of the cubic structure in the temperature interval 150-280 K with $a_o = a_1 + a_2\Theta_s\coth\left(\frac{\Theta_s}{T}\right)$, where Θ_s is a saturation temperature (Carpenter 2007; Salje *et al*. 1991; Meyer *et al*. 2000, 2001; Sondergeld *et al*. 2000), and then extrapolating this to temperatures below T_V.

$Fd\bar{3}m \rightarrow Cc$	$Fd\bar{3}m \rightarrow$ rhombohedral
$e_1 + e_2 = \frac{a/\sqrt{2} - a_o}{a_o} + \frac{b/\sqrt{2} - a_o}{a_o}$	$e_1 = e_2 = e_3 = \frac{a - a_o}{a_o}$
$e_3 = \frac{c\sin\beta^*/2 - a_o}{a_o} \approx \frac{c/2 - a_o}{a_o}$	$e_4 = e_5 = e_6 = \frac{a}{2a_o}\cos\alpha^* \approx \frac{1}{2}\cos\alpha^*$
$e_6 = \frac{a/\sqrt{2} - a_o}{a_o} - \frac{b/\sqrt{2} - a_o}{a_o}$	
$e_4 = e_5 = \frac{1}{2}\frac{c/2}{a_o}\cos\beta \approx \frac{1}{2}\cos\beta$	

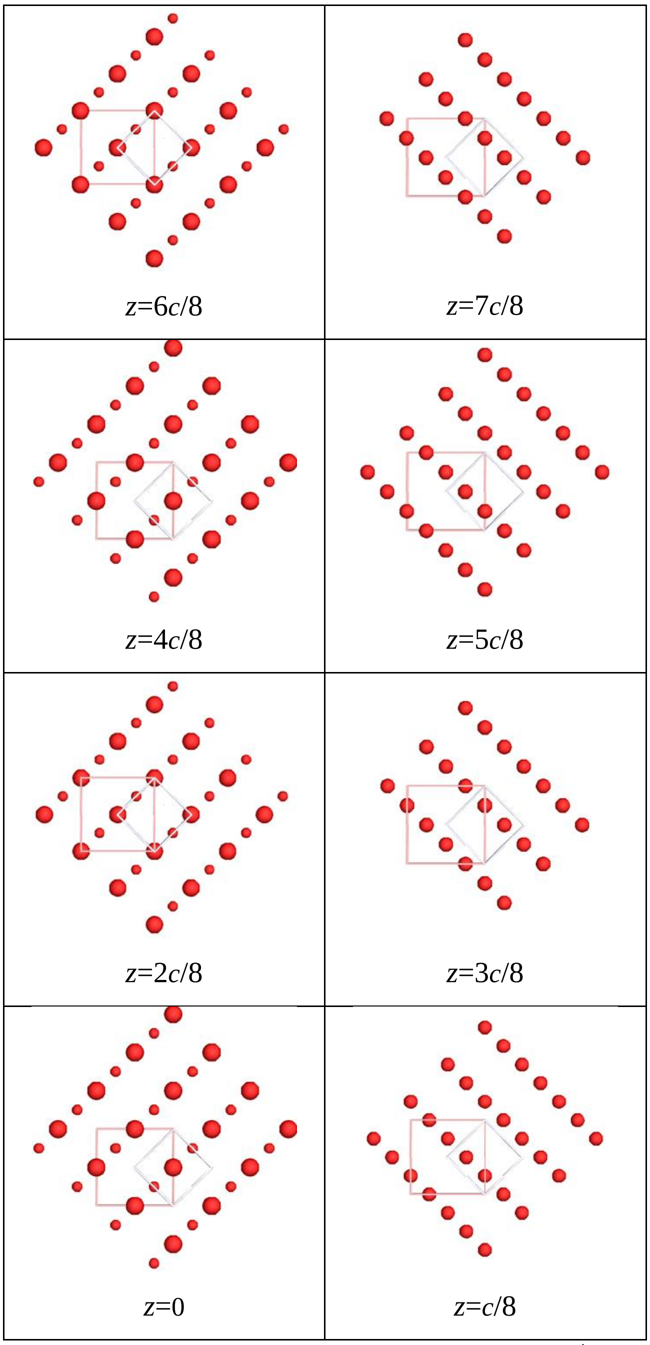

Figure S1. Ordering scheme with symmetry of irrep Γ_5^+ for cations on octahedral sites of the spinel structure, as generated using ISOVIZ for the structure in space group *P*2/*c*. Two different octahedral cations are shown as large and small circles in 8 layers perpendicular to the c-axis. Circles with intermediate size represent cation sites at which there are no changes due to operation of the irrep. For the sake of clarity, oxygen atoms and tetrahedral cations are not shown. The parent F-cell is outlined in pink and the product P-cell in grey.

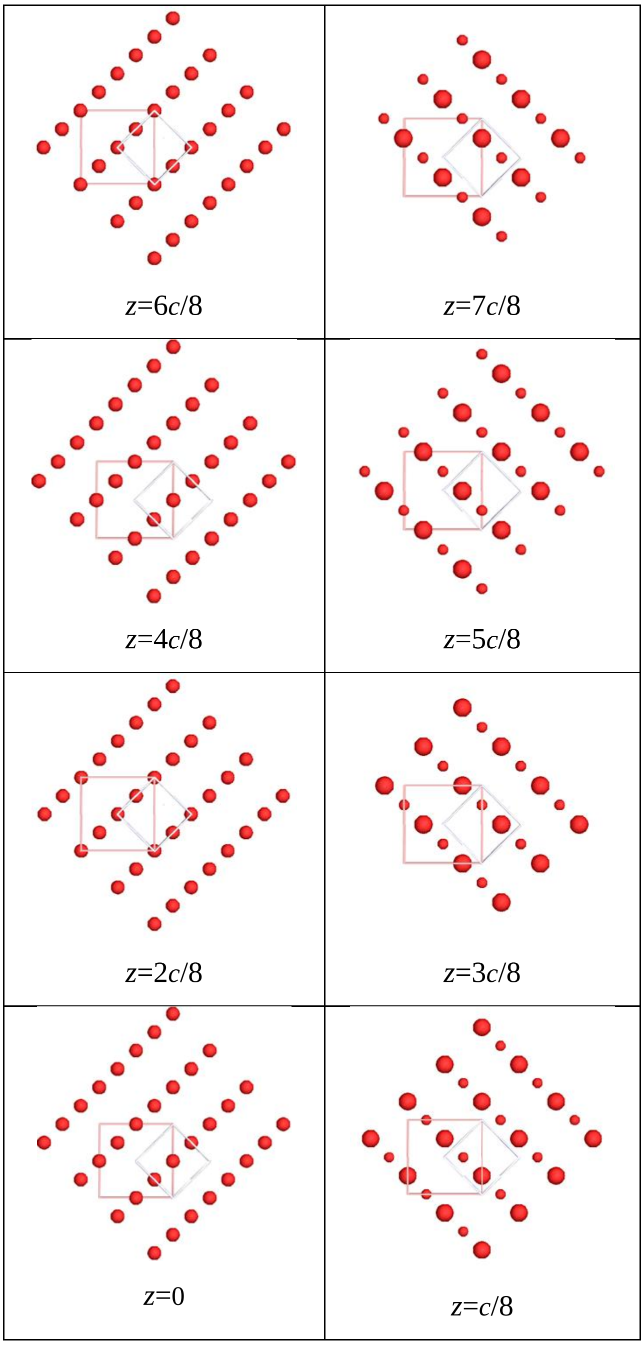

Figure S2. Ordering scheme with symmetry of irrep Δ_5 for cations on octahedral sites of the spinel structure, as generated using ISOVIZ for the structure in space group *P*2/*c*. Two different octahedral cations are shown as large and small circles in 8 layers perpendicular to the c-axis. Circles with intermediate size represent cation sites at which there are no changes due to operation of the irrep. For the sake of clarity, oxygen atoms and tetrahedral cations are not shown. The parent F-cell is outlined in pink and the product P-cell in grey.

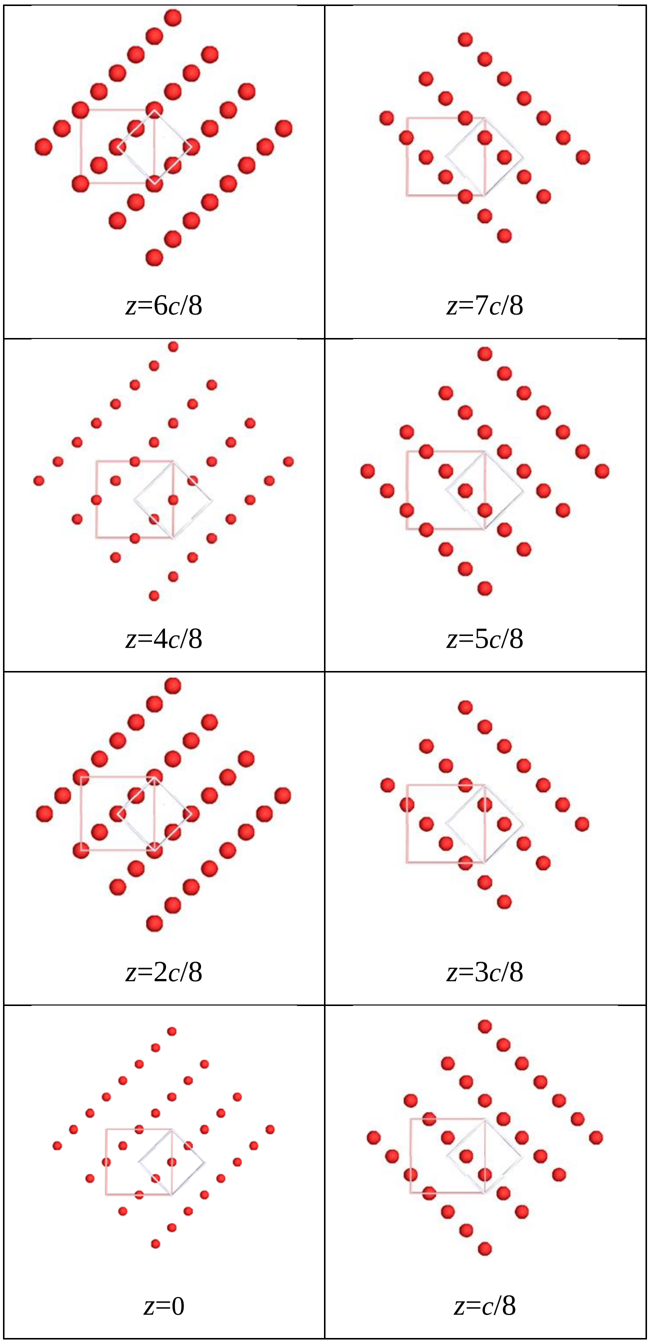

Figure S3. Ordering scheme with symmetry of irrep X_1 for cations on octahedral sites of the spinel structure, as generated using ISOVIZ for the structure in space group *P*2/*c*. Two different octahedral cations are shown as large and small circles in 8 layers perpendicular to the c-axis. Circles with intermediate size represent cation sites at which there are no changes due to operation of the irrep. For the sake of clarity, oxygen atoms and tetrahedral cations are not shown. The parent F-cell is outlined in pink and the product P-cell in grey.

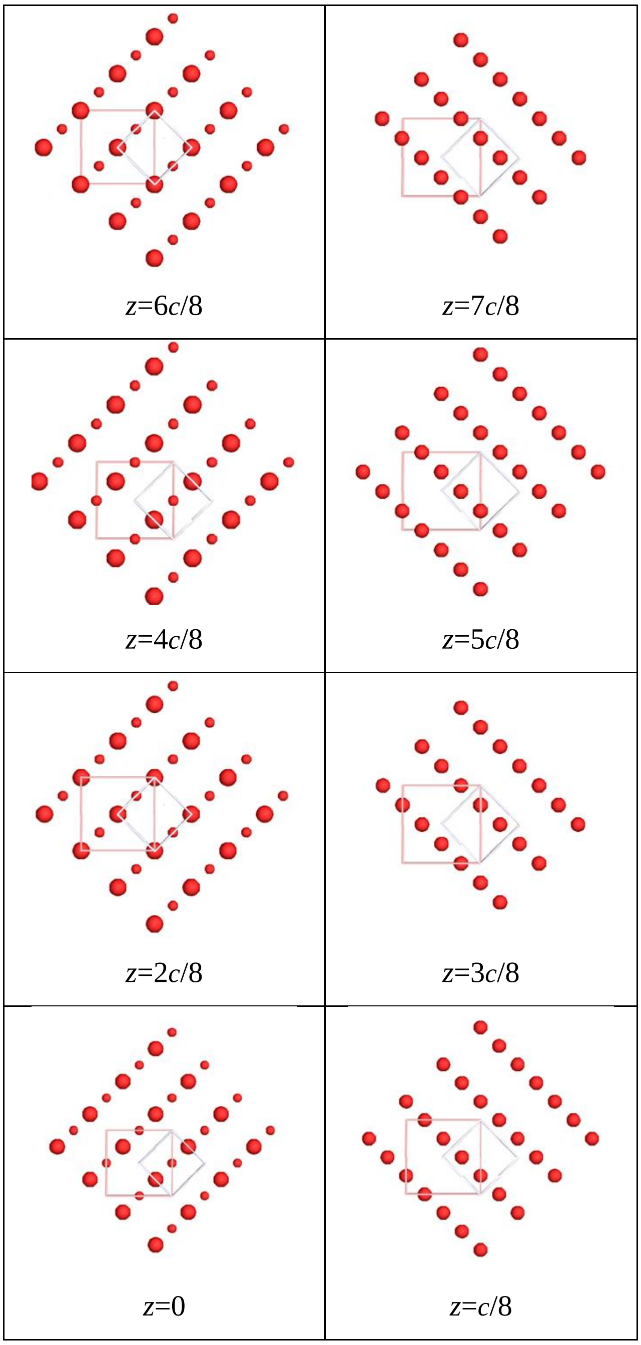

Figure S4. Ordering scheme with symmetry of irrep X_3 for cations on octahedral sites of the spinel structure, as generated using ISOVIZ for the structure in space group $P2/c$. Two different octahedral cations are shown as large and small circles in 8 layers perpendicular to the c-axis. Circles with intermediate size represent cation sites at which there are no changes due to operation of the irrep. For the sake of clarity, oxygen atoms and tetrahedral cations are not shown. The parent F-cell is outlined in pink and the product P-cell in grey.

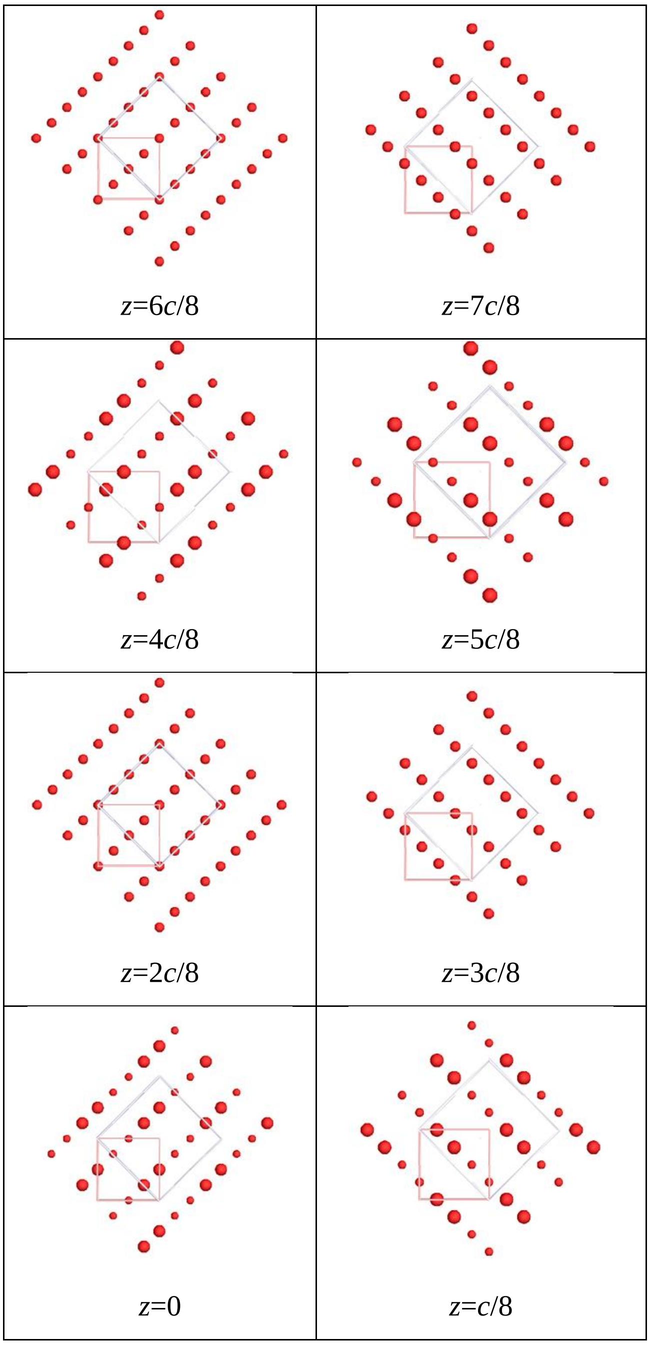

Figure S5. The first ordering scheme with symmetry of irrep W1 for cations on octahedral sites of the spinel structure, as generated using ISOVIZ for the structure in space group *Cc*. Two different octahedral cations are shown as large and small circles in 8 layers perpendicular to the c-axis. Circles with intermediate size represent cation sites at which there are no changes due to operation of the irrep. For the sake of clarity, oxygen atoms and tetrahedral cations are not shown. The parent F-cell is outlined in pink and the product C-cell in grey.

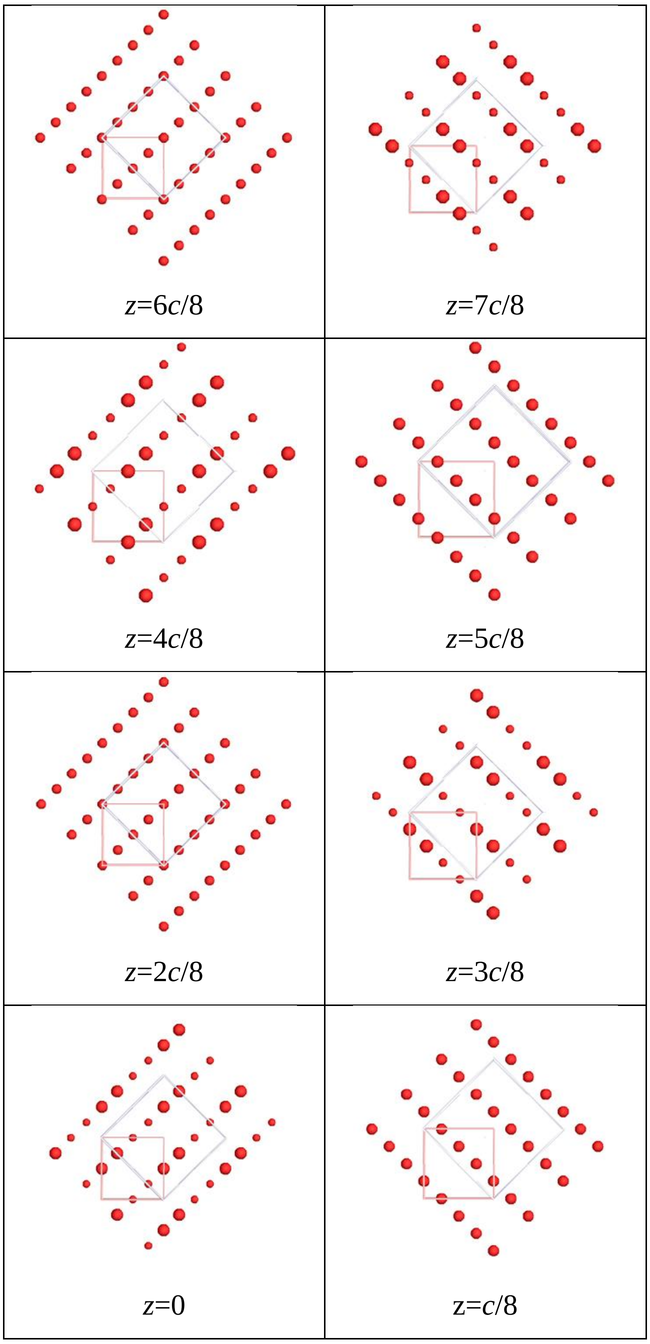

Figure S6. The second ordering scheme with symmetry of irrep W1 for cations on octahedral sites of the spinel structure, as generated using ISOVIZ for the structure in space group *Cc*. Two different octahedral cations are shown as large and small circles in 8 layers perpendicular to the c-axis. Circles with intermediate size represent cation sites at which there are no changes due to operation of the irrep. For the sake of clarity, oxygen atoms and tetrahedral cations are not shown. The parent F-cell is outlined in pink and the product C-cell in grey.

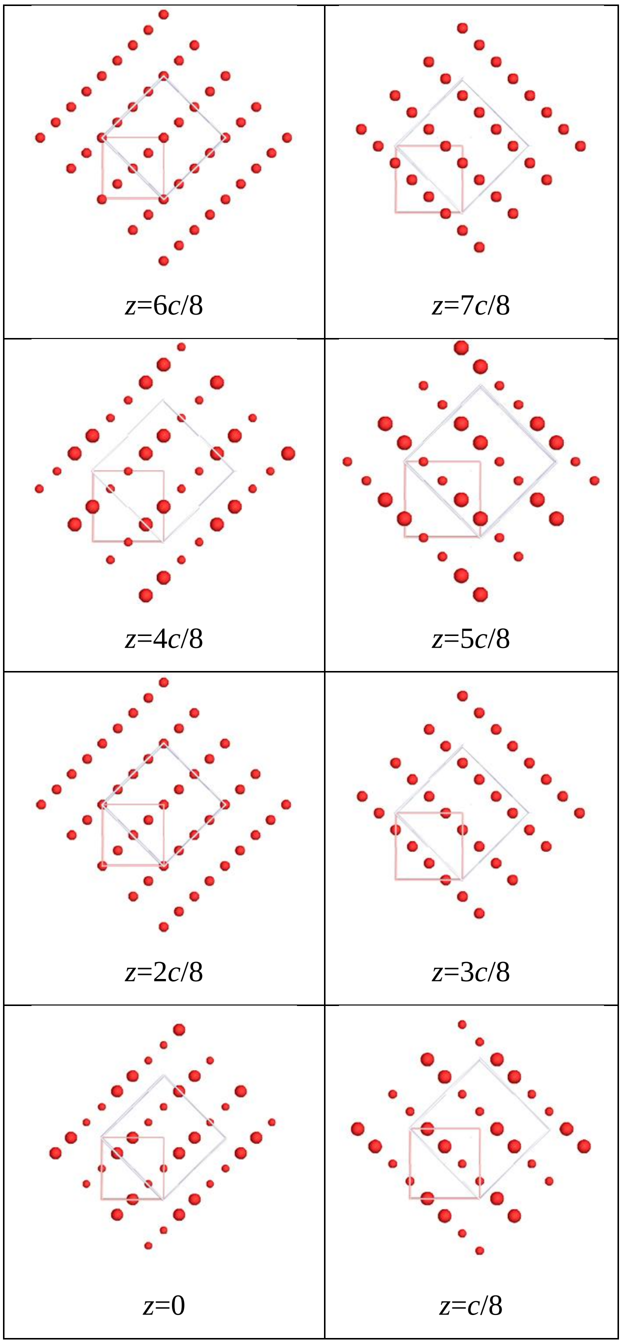

Figure S7. The first ordering scheme with symmetry of irrep W2 for cations on octahedral sites of the spinel structure, as generated using ISOVIZ for the structure in space group *Cc*. Two different octahedral cations are shown as large and small circles in 8 layers perpendicular to the c-axis. Circles with intermediate size represent cation sites at which there are no changes due to operation of the irrep. For the sake of clarity, oxygen atoms and tetrahedral cations are not shown. The parent F-cell is outlined in pink and the product C-cell in grey.

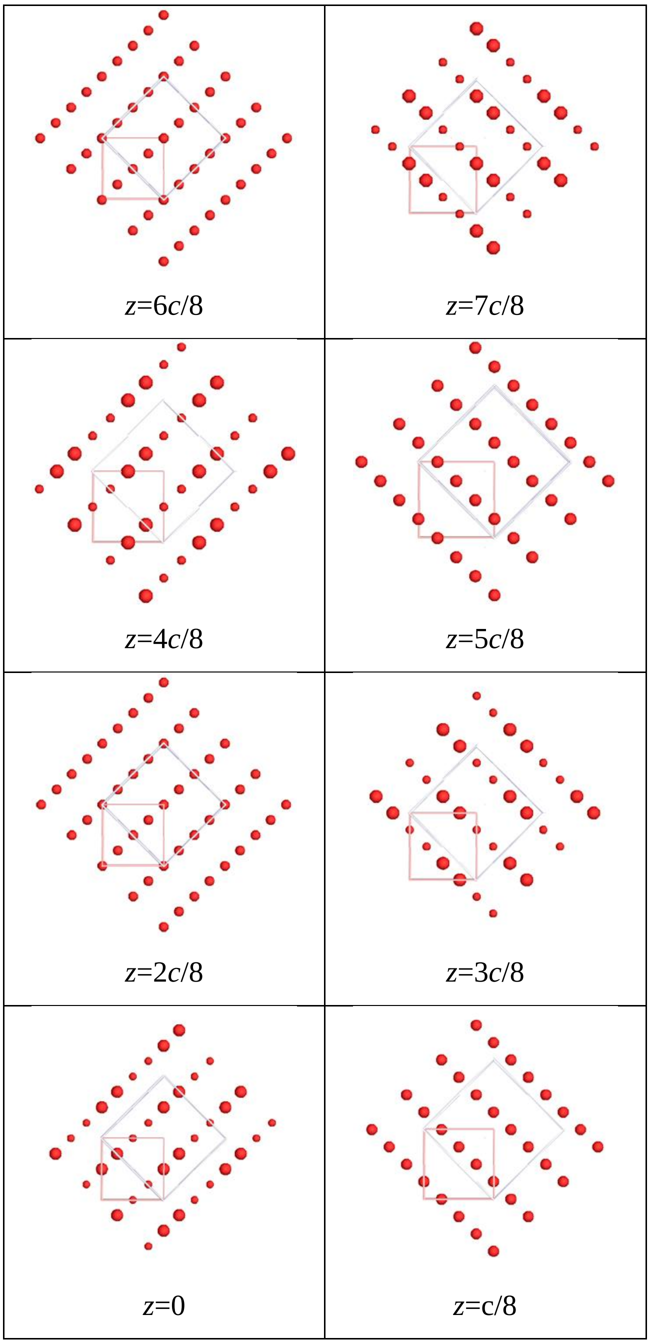

Figure S8. The second ordering scheme with symmetry of irrep W2 for cations on octahedral sites of the spinel structure, as generated using ISOVIZ for the structure in space group *Cc*. Two different octahedral cations are shown as large and small circles in 8 layers perpendicular to the c-axis. Circles with intermediate size represent cation sites at which there are no changes due to operation of the irrep. For the sake of clarity, oxygen atoms and tetrahedral cations are not shown. The parent F-cell is outlined in pink and the product C-cell in grey.

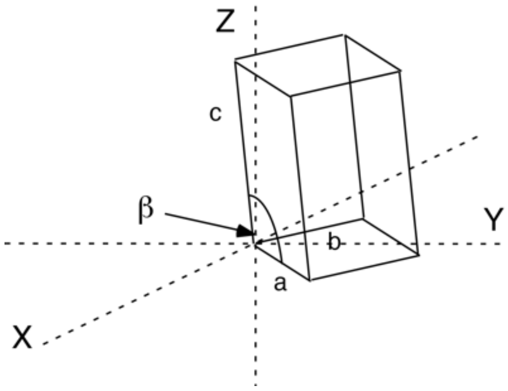

Figure S9. Setting for the unit cell of the *Cc* structure with respect to reference axes, using basis (1,1,0), (-1,1,0), (0,0,2) and origin (0,0,1/2). Setting for *P*2/*c*: basis (1/2,1/2,0), (-1/2,1/2,0), (0,0,2), origin (0,0,0).

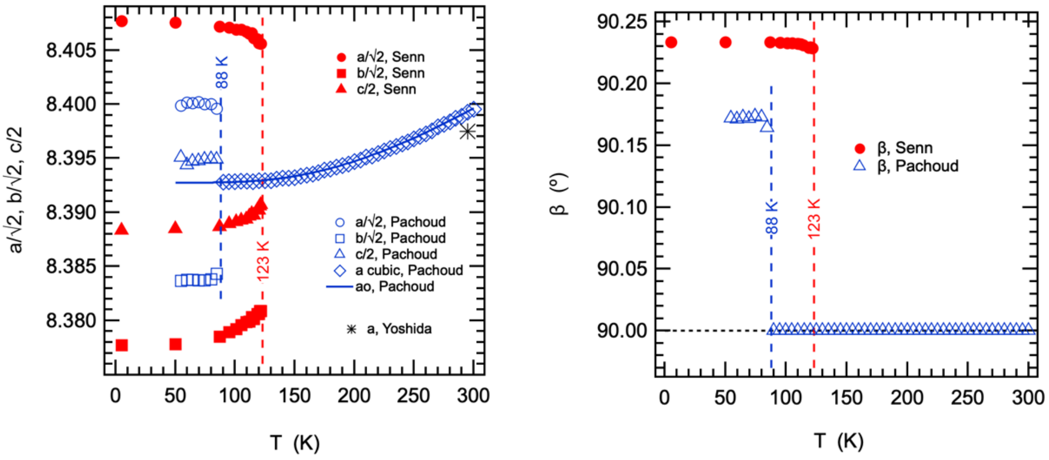

Figure S10. Lattice parameter data from Figure 3 of Senn *et al*. (2015) for a synthetic magnetite sample with T_V = 122 ± 1 K (Powder X-ray diffraction data refined in space group *Cc*). Values of a_o were taken as $a_o = \left(\frac{abc\sin\beta}{4}\right)^{1/3}$. Red broken lines are at 123 K. The curved line is a fit of $a_o = a_1 + a_2\Theta_s\coth\left(\frac{\Theta_s}{T}\right)$ to data for *a* of the sample of Pachoud *et al*. (2020) in the temperature interval 90-325 K (a_1 = 8.3599Å, a_2 = 9.2909x10^{-5} Å.K^{-1}, Θ_s = 353.47 K). Also included is the cubic lattice parameter at 295 K for a synthetic magnetite sample with T_v = 121 K from Yoshida and Iida (1979). Blue broken lines are at 88 K.

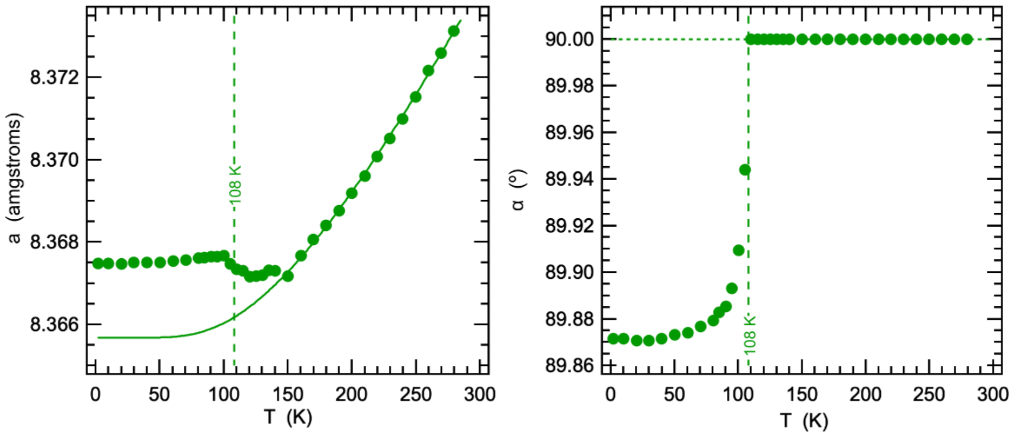

Figure S11. Lattice parameter data from Figure 3 of Wright *et al*. (2000) for a synthetic magnetite sample with T_V = 110 ± 5 K (Powder neutron diffraction data refined with rhombohedral lattice geometry). The curved line is a fit of $a_o = a_1 + a_2\Theta_s\coth\left(\frac{\Theta_s}{T}\right)$ to data for a in the temperature interval 150-279 K (a_1 = 8.3515 Å, a_2 = 6.4672 x 10^{-5} Å.K^{-1}, Θ_s = 219.82 K).
